# Antioxidative and Anti-Inflammatory Activities of Rosebud Extracts of Newly Crossbred Roses

**DOI:** 10.3390/nu15102376

**Published:** 2023-05-19

**Authors:** Cuicui Wang, In-Jeong Kim, Hye-Rim Seong, Chan Ho Noh, Sangryong Park, Tae Myoung Kim, Heon Sang Jeong, Ka Young Kim, Seung Tae Kim, Hyun-Gyun Yuk, Sang-Chul Kwon, Ehn-Kyoung Choi, Yun-Bae Kim

**Affiliations:** 1 College of Veterinary Medicine, Chungbuk National University, Cheongju 28644, Republic of Korea; 2 Central Research Institute, Designed Cells Co., Ltd., Cheongju 28576, Republic of Korea; 3 Department of Food Science and Biotechnology, Chungbuk National University, Cheongju 28644, Republic of Korea; 4 Gumi Floriculture Research Institute, Gyeongsanbuk-do Agricultural Research & Extension Services, Gumi 39102, Republic of Korea; 5 Department of Food Science and Biotechnology, Korea National University of Transportation, Jeungpyeong 27909, Republic of Korea

**Keywords:** rosebud extract, Pretty Velvet, polyphenol, antioxidation, anti-inflammation, nitric oxide, prostaglandin E_2_

## Abstract

Oxidative stress and inflammation are basic pathogenic factors involved in tissue injury and pain, as well as acute and chronic diseases. Since long-term uses of synthetic steroids and non-steroidal anti-inflammatory drugs (NSAIDs) cause severe adverse effects, novel effective materials with minimal side effects are required. In this study, polyphenol content and antioxidative activity of rosebud extracts from 24 newly crossbred Korean roses were analyzed. Among them, Pretty Velvet rosebud extract (PVRE) was found to contain high polyphenols and to show in vitro antioxidative and anti-inflammatory activities. In RAW 264.7 cells stimulated with lipopolysaccharide (LPS), PVRE down-regulated mRNA expression of inducible nitric oxide synthase (iNOS) and cyclooxygenase-2 (COX-2), and thereby decreased nitric oxide (NO) and prostaglandin E_2_ (PGE_2_) production. In a subcutaneous air-pouch inflammation model, treatment with PVRE decreased λ-carrageenan-induced tissue exudation, infiltration of inflammatory cells, and inflammatory cytokines such as tumor necrosis factor-α and interleukin-1β concentrations, as achieved with dexamethasone (a representative steroid). Notably, PVRE also inhibited PGE_2_, similar to dexamethasone and indomethacin (a representative NSAID). The anti-inflammatory effects of PVRE were confirmed by microscopic findings, attenuating tissue erythema, edema, and inflammatory cell infiltration. These results indicate that PVRE exhibits dual (steroid- and NSAID-like) anti-inflammatory activities by blocking both the iNOS—NO and COX-2—PG pathways, and that PVRE could be a potential candidate as an anti-inflammatory material for diverse tissue injuries.

## 1. Introduction

Oxidation and oxidative phosphorylation are essential processes in our body for metabolism and energy formation, but oxidative stress is a negative reaction of the imbalance between oxidation and antioxidation caused by the over-production of reactive oxygen species (ROS). Excessive production of ROS attacks proteins, lipids, and nucleic acids, resulting in cellular disruption, immune reaction, and inflammation [1,2].

Inflammation is the complex biological response of vascular tissues to toxicants, such as erythema and edema, which are induced by enhanced vascular permeability at the injury sites, resulting in the extravasation of blood macromolecules [3]. Inflammation is a protective attempt of the body to eliminate harmful stimuli and initiate a healing process [4]. However, if the host fails to eliminate the stimuli early enough or undergoes an improper recovery process, the unresolved inflammation may cause tissue and organ dysfunctions [5]. Inflammation underlies diverse physiological and pathological processes [6]. Previous reports have provided evidence that inflammation plays a major role in the pathogenesis of acute and chronic diseases [7,8]. Thus, the management of inflammation is essential to attenuate pain, reduce cell and tissue injury, and improve patients’ quality of life.

As an in vitro inflammation model, RAW 264.7 cells, a macrophage cell line, stimulated with lipopolysaccharide (LPS), are commonly used [9,10] since during activation of the inflammatory cells, two major inflammatory mediators such as nitric oxide (NO) and prostaglandin E_2_ (PGE_2_) are released [11]. In brief, dual inflammatory pathways are summarized as follows: (1) Tumor-necrosis factor-α (TNF-α)–inducible nitric oxide synthase (iNOS)–NO pathway: Inflammatory cells stimulated by TNF-α express iNOS producing NO, which is inhibited by corticosteroids. (2) Arachidonic acid (AA)–cyclooxygenase-2 (COX-2)–PGE_2_ pathway: Activated inflammatory cells express COX-2 that degrades AA producing PGE_2_, which is inhibited by NSAIDs [12]. Therefore, for the screening of anti-inflammatory compounds, the mRNA expression of iNOS and COX-2, as well as the production of NO and PGE_2,_ can be analyzed to find out their action mechanisms, that is, steroid-like and/or NSAID-like activities.

Among many in vivo skin inflammation models, λ-carrageenan-induced air-pouch inflammation is recommended, in which exudate can be easily collected for the analysis of inflammatory cells and mediators in the exudate. Carrageenans are high-molecular-weight sulfated polygalactans obtained from marine algae [13]. Air pouches can be produced by subcutaneously injecting air into the backside of rodents, and λ-carrageenan injection into the pouch induces inflammation [14,15]. That is, the pathogenic agent λ-carrageenan induces inflammatory responses in the lining tissue of the pouch wall [16], leading to the extravasation of blood cells and fluid into the pouch. Investigators have shown the accumulation of inflammatory cells, including macrophages, white blood cells (WBCs), and lymphocytes, cytokines such as TNF-α and interleukin-1β (IL-1β), as well as mediators such as NO and PGE_2_ [8,17,18]. These endpoints can be quantified and used to determine the degree of inflammation, resolution of inflammation, or anti-inflammatory and antioxidative activities of test compounds, since TNF-α and IL-1β are the main inflammatory cytokines triggering activation of inflammatory cells, and NO and PGE_2_ are major inflammatory mediators causing pain and tissue responses including vasodilatation, erythema, and edema [19].

It is commonly known that TNF-α–iNOS–NO and AA–COX-2–PGE_2_ inflammatory pathways are blocked by steroids and NSAIDs, respectively [12]. However, severe adverse effects of such drugs are easily developed [20,21,22]. Indeed, it is well known that high doses and/or long-term use of synthetic steroids cause tolerance, glaucoma, cataracts, diabetes, osteoporosis, and muscle weakness [22]. NSAIDS can induce gastrointestinal bleeding, congestive heart failure, chronic kidney diseases with renal papillary necrosis, and allergic reactions. Thus, therapeutic options need better outcomes with fewer side effects, and research with natural sources is a good path to explore [23,24].

Roses are dicotyledons belonging to the genus Rosa in the family Rosacea. Traditionally, roses are well known to contain bioactive ingredients, especially volatile molecules. So, rose oils have been used for perfumes, cosmetics, and foods [25]. Rose has been confirmed to contain a large amount of antioxidants, and is used as an edible flower in many countries, as well as a cosmetic ingredient [26]. It was found that rose petals contained antioxidant ingredients, including vitamins, polyphenols, anthocyanins, flavonoids, and lactones, and thus displayed antioxidative activity higher than other plants [27,28] comparable to that of butylated hydroxytoluene (BHT), a representative synthetic antioxidant [29,30,31]. Related to effectiveness, anthocyanins of rose flowers have been reported to be effective in preventing cardiovascular diseases, cancers, and diabetes [32]. In many of our studies, it has been confirmed that rosebud extracts have strong antimicrobial potential [33,34] and exhibit anti-allergic, anti-atopic, and anti-inflammatory activities [35,36], and antioxidation and anti-inflammation-related neuroprotective effects [37,38].

Therefore, the purpose of this study is to screen novel functional materials with high antioxidative and anti-inflammatory activities. In this context, polyphenol contents and antioxidative activities of 24 rosebud extracts from newly crossbred roses were assessed first, and the anti-inflammatory effects of a selected candidate were confirmed in vitro and in vivo using RAW 264.7 macrophages and a skin inflammation model, respectively.

## 2. Materials and Methods

### 2.1. Extraction of Rosebuds

This study used dried rosebuds from 24 newly crossbred Korean roses (*Rosa hybrida*) from Gumi Floriculture Research Institute (Gumi, Republic of Korea) in 2021, which are (1) Lover Shy, (2) Lovely Scarlet, (3) Loving Heart, (4) Red Perfume, (5) Luminus, (6) Mirinae Gold, (7) Betty, (8) Bichina, (9) Aileen, (10) Onnuri, (11) Yunina, (12) Jaemina Red, (13) Jinseonmi, (14) Chilbaegri, (15) Tamina, (16) Tamnari, (17) Pretty Velvet, (18) Peach Grace, (19) Pink Love, (20) Pink Perfume, (21) Hanaro, (22) Hanaram, (23) Hanggina, and (24) Ice Wing. Since total rosebud extracts were higher in antioxidative activity than petal extracts, in addition to having higher yield, we used total rosebuds [38].

Based on our previous study, the rosebuds were extracted with 80% ethanol to achieve high polyphenol content [39]). The dried rosebuds were pulverized in a rotor mill (Laval Lab Inc., Laval, QC, Canada), immersed in 80% ethanol in an ultrasonic water bath, heated at 60~70 °C for 2 h, and then ultrasonic-extracted for 1 h. Actually, the extraction solvent/solid ratio was set to 49:1 (980 mL 80% ethanol/20 g dried rosebuds). After extraction, the mixture was cooled, filtered, and then concentrated under a reduced pressure to 50 brix using a vacuum evaporator (Rotary Vacuum Evaporator N-N series; Eyela, Tokyo, Japan), and then used as a test sample.

### 2.2. Analysis of Antioxidant Ingredients

Total polyphenols as a major antioxidant component of plants were analyzed as described previously [40]. That is, Folin–Ciocalteu’s phenol reagent was reduced by polyphenolic compounds to develop a color of molybdenum. The sample concentration was adjusted to 1 mg/mL. A total of 2 mL Na_2_CO_3_ (2%) was added into 100 μL sample solution and incubated for 3 min. After adding 100 μL Folin–Ciocalteu’s reagent (50%) and reacting for 30 min, the absorbance was measured at 750 nm using a spectrophotometer (UV-1650, Shimadzu Corporation, Kyoto, Japan). A standard calibration curve was produced with gallic acid (Sigma-Aldrich, St. Louis, MO, USA) as a standard compound diluted 10, 20, 30, 40, and 50 times, and the polyphenol contents were expressed as mg gallic acid equivalent (GAE) in 1 g extract.

### 2.3. Measurement of Antioxidative Activities

#### 2.3.1. Analysis of ABTS-Scavenging Activity

The antioxidative efficacy of rosebud extracts was measured by the scavenging potential of 2,2-azino-bis (3-ethylbenzothiazoline 6-sulfonic acid) (ABTS, Sigma-Aldrich) [41]. A stock ABTS radical cation solution (7 mM) was added to a 2.45 mM potassium and sulfate solution and stirred well for 12 to 16 h in a dark room. The solution was diluted with distilled water (DW) to adjust absorbance of 1.4 to 1.5 at 735 nm. Diluted 1 mL ABTS solution was added to 50 μL test substance or DW (blank) to adjust to 0.5 mg/mL concentration. After 1 h, the absorbance was measured at 735 nm. ABTS radical-scavenging activity, i.e., electron-donating ability, was expressed as mg ascorbic acid equivalent (AAE) in 1 g extract with the difference in absorbance according to the addition and non-addition (DW) of the test substance.

#### 2.3.2. Analysis of DPPH-Scavenging Activity

Additional antioxidative efficacy of the rosebud extracts was measured as described previously [42], based on the scavenging potential of 1,1-diphenyl-2-picrylhydrazyl (DPPH, Sigma-Aldrich). That is, 0.2 mL rose extract or DW (blank) was added into 0.8 mL DPPH solution (0.2 mM) to adjust to 0.1 mg/mL. After 30 min incubation, absorbance was measured at 520 nm. DPPH radical-scavenging activity, i.e., electron-donating ability, was expressed as mg AAE in 1 g extract with the difference in absorbance according to the addition and non-addition (DW) of the test substance.

#### 2.3.3. Analysis of Correlation between Antioxidant Contents and Antioxidative Activities

The correlations between antioxidant (polyphenol) contents and antioxidative activities were analyzed. Linear regression coefficients were calculated for the estimation of relationship.

### 2.4. Measurement of Anti-Inflammatory Activities

#### 2.4.1. Analysis of NO-Inhibitory Activity

RAW 264.7 cells were from American Type Culture Collection (ATCC, Manassas, VA, USA). Cells were cultivated with Dulbecco’s Modified Eagle’s Medium (DMEM, Biowest, Kansas City, MO, USA) supplemented with 10% fetal bovine serum (FBS, Biowest).

RAW 264.7 cells were seeded in a 96-well plate at a density of 5 × 10^5^ cells/mL (0.1 mL/well). The cells were incubated with LPS (Sigma-Aldrich, 1 μg/mL) and 100 μg/mL of each of 24 rosebud extracts for 24 h. The cell culture supernatants were harvested, and the concentration of NO was quantified by Griess reagent system (Promega, Madison, WI, USA).

#### 2.4.2. Analysis of Correlation between Antioxidant Contents and NO-Inhibitory Activities

The correlations between antioxidant (polyphenol) contents and NO-inhibitory activity of 24 rosebud extracts were analyzed. Linear regression coefficient was calculated for the estimation of relationship.

Based on the antioxidant contents, antioxidative activities, and NO-inhibitory potentials of 24 rosebud extracts, Pretty Velvet rosebud extract (PVRE) was selected as a candidate for further studies on anti-inflammatory efficacies in vitro and in vivo, and its underlying mechanisms.

### 2.5. Measurement of In Vitro Anti-Inflammatory Activity of PVRE

#### 2.5.1. MTT Assay for Cytotoxicity

3-(4,5-Dimethylthiazol-2-yl)-2,5-diphenyl-^2^H-tetrazolium bromide (MTT) was purchased from Sigma-Aldrich (98% purity). Briefly, MTT powder was dissolved in Dulbecco’s phosphate-buffered saline (D-PBS, Biowest) to a final concentration of 5 mg/mL (MTT stock solution).

RAW 264.7 cells were seeded in a 96-well plate at a density of 5 × 10^5^ cells/mL (0.1 mL/well). The cells were incubated with various concentrations (10, 30, or 100 μg/mL) of PVRE for 24 h. A total of 100 μL of DMEM containing 10% MTT stock solution (MTT medium) was added into each cell culture well (0.1 mL/well) and incubated under 5% CO_2_ at 37 °C for a further 1 h. After discarding the MTT medium, the formazan produced in the cells was extracted with 100 μL dimethylsulfoxide (DMSO). The absorbance was read at 540 nm.

#### 2.5.2. qPCR Analysis of mRNA Expressions of iNOS and COX-2

RAW 264.7 cells were seeded into a 24-well plate (5 × 10^5^ cells/mL) and incubated under 5% CO_2_ at 37 °C overnight. The cells were incubated with LPS (1 μg/mL) and various concentrations (10, 30, or 100 μg/mL) of PVRE for 24 h. Total RNA was extracted using 1 mL of RNAiso PLUS (TaKaRa, Kusatsu, Japan). Then, 1 μg of total RNA was reverse-transcribed to cDNA. Real-time qPCR samples were prepared with a PrimeScript^TM^ RT reagent Kit with gDNA Eraser (TaKaRa). The primer sequences for iNOS, COX-2, and GAPDH are described in Table 1. qPCR was performed with an AriaMx Real-Time PCR system (Agilent Technologies, Santa Clara, CA, USA) using a two-step protocol (40 cycles of 95 °C for 30 s followed by 60 °C for 30 s).

#### 2.5.3. Chemical Analysis of NO and PGE_2_

After collection of RAW 264.7 cells for the analysis of mRNA expression, the culture supernatant was harvested. The concentrations of NO and PGE_2_ were measured using the Griess reagent and enzyme-linked immunosorbent assay (ELISA) kit (R&D Systems, Minneapolis, MN, USA), respectively.

### 2.6. Measurement of In Vivo Anti-Inflammatory Activity of PVRE

#### 2.6.1. Animals

Six-week-old male Sprague Dawley rats were purchased from DBL (Eumsung, Republic of Korea). The animals were housed in a room with a constant temperature (23 ± 2 °C), relative humidity (55 ± 10%), and 12 h light/dark cycle, and fed with standard rodent chow and purified water ad libitum.

All animal experimental procedures were approved and carried out in accordance with the Institutional Animal Care and Use Committee of Laboratory Animal Research Center at Chungbuk National University, Korea (Approval No. CBNUR-1629-21).

#### 2.6.2. Experimental Design Using an Air-Pouch Inflammation Model

An air-pouch model of λ-carrageenan-induced skin inflammation was established in rats. Briefly, rats (*n* = 8/group) were anesthetized with isoflurane. Then, 20 mL of sterile air was injected subcutaneously into the mid-back side using a syringe to make an air pouch. On the 5th and 6th days, 10 mL sterile air was re-injected to maintain the size of the pouch. Twenty-four hours later, inflammation was induced by injecting 1 mL λ-carrageenan solution (2% in D-PBS) into the pouch. PVRE (10, 30, or 100 mg/kg), dexamethasone (Sigma-Aldrich; 2 mg/kg) or indomethacin (Sigma-Aldrich; 2 mg/kg) were administrated intraperitoneally 30 min before λ-carrageenan injection. Six hours after λ-carrageenan injection, the exudate in the pouch was collected. An aliquot of the exudate was analyzed for inflammatory cells. Remaining exudate was centrifuged at 10,000× *g* at 4 °C for 10 min, and the supernatant was used for the analysis of inflammatory mediators. The centrally positioned skin tissues of the pouches were excised for ImageJ 1.54d (Java 1.8.0_345, 64-bit; NIH, Bethesda, MD, USA) analysis and histopathological examination.

#### 2.6.3. Analysis of Exudate Volume and Inflammation Cells

After sacrifice of the rats, the air-pouch exudate was collected. Briefly, 2 mL cold saline (0.9% NaCl) was administered into the pouch and gently massaged. All the fluid was harvested using a syringe, the volume was recorded, and net exudate was calculated by subtracting 2 mL saline injected.

The exudate was analyzed for total white blood cells (WBCs), neutrophils, monocytes, and lymphocytes with a hematology analyzer (IDEXX Procyte Dx, Westbrook, CT, USA).

#### 2.6.4. ELISA Analysis of Inflammatory Cytokines

The levels of TNF-α and IL-1β in air-pouch exudate were measured using ELISA kits (R&D Systems) according to the manufacturer’s instructions.

#### 2.6.5. Chemical Analysis of NO and PGE_2_

The concentrations of NO and PGE_2_ in air-pouch exudate were quantified using the Griess reagent (Sigma-Aldrich) and ELISA kit (R&D Systems), respectively, as described above.

#### 2.6.6. ImageJ Analysis of Dermal Blood Vessel Area

After the rats were sacrificed, the back skin was cut off in the same area and photographed, and the area of blood vessels on the back skin was observed and calculated by ImageJ software (NIH).

#### 2.6.7. Microscopic Examination of Tissue Inflammation

Skin tissues obtained from the pouches were fixed with 10% neutral formalin, followed by tissue processing and paraffin embedding. Paraffin blocks were sectioned (4 μm in thickness) and stained with hematoxylin-eosin. Histopathological examination was then performed under a light microscope (Nikon, Tokyo, Japan).

### 2.7. Statistical Analysis

The data were described as mean ± standard error. Statistical significance between the groups was analyzed by one-way analysis of variance using the SPSS statistical software (SPSS Inc., Chicago, IL, USA). *p*-values of less than 0.05 were considered statistically significant.

## 3. Results

### 3.1. Antioxidant Contents in 24 Rosebud Extracts

**Figure 1:**
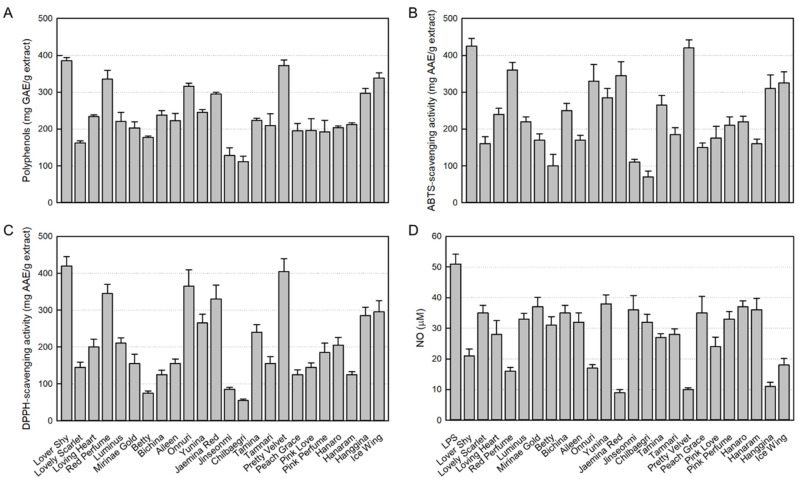
Antioxidant contents in 24 rosebud extracts and their antioxidative and anti-inflammatory activities (100 μg/mL). (**A**) Polyphenol contents. (**B**) 2,2-Azino-bis(3-ethylbenzothiazoline 6-sulfonic acid) (ABTS) radical-scavenging activity. (**C**) 1,1-Diphenyl-2-picrylhydrazyl (DPPH) radical-scavenging activity. (**D**) Inhibitory activity on nitric oxide (NO) production from RAW 264.7 macrophages stimulated with lipopolysaccharide (LPS, 1 μg/mL).

As a major antioxidant ingredient, the contents of polyphenols in 24 rosebud extracts tested were in the range of 111.7 to 385.5 mg GAE/g extract. In comparison, Lover Shy (385.5 ± 0.8 mg GAE/g extract), Pretty Velvet (372.5 ± 1.5 mg GAE/g extract), Ice Wing (338.4 ± 1.3 mg GAE/g extract), Red Perfume (335.2 ± 2.4 mg GAE/g extract), Onnuri (316.2 ± 0.8 mg GAE/g extract), Hanggina (296.6 ± 1.3 mg GAE/g extract), and Jaemina Red (295.2 ± 0.1 mg GAE/g extract) showed relatively high polyphenol contents (Figure 1A). Especially, Lover Shy and Pretty Velvet extracts were found to contain the highest antioxidant concentrations.

### 3.2. Antioxidant Activities of 24 Rosebud Extracts

As shown in Figure 1B, among the 24 rosebud extracts tested (100 μg/mL), high ABTS radical-scavenging activities were confirmed in Lover Shy (425.0 ± 21.0 mg/AAE/g extract), Pretty Velvet (420.2 ± 22.3 mg/AAE/g extract), Red Perfume (360.1 ± 20.7 mg/AAE/g extract), Jaemina Red (345.4 ± 38.2 mg/AAE/g extract), Onnuri (330.3 ± 45.2 mg/AAE/g extract), Ice Wing (325.3 ± 29.9 mg/AAE/g extract), and Hanggina (310.0 ± 36.8 mg/AAE/g extract).

Similarly, high DPPH radical-scavenging activities were also observed in Lover Shy (420.3 ± 24.8 mg/AAE/g extract), Pretty Velvet (405.4 ± 35.2 mg/AAE/g extract), Onnuri (365.4 ± 43.9 mg/AAE/g extract), Red Perfume (345.1 ± 24.6 mg/AAE/g extract), Jaemina Red (330.2 ± 38.0 mg/AAE/g extract), Ice Wing (295.4 ± 30.7 mg/AAE/g extract), and Hanggina (285.1 ± 23.3 mg/AAE/g extract) (Figure 1C).

Collectively, Lover Shy and Pretty Velvet were found to have the highest antioxidant activities against ABTS and DPPH.

### 3.3. Inhibitory Activities of 24 Rosebud Extracts on NO Production

NO produced from LPS-stimulated RAW 264.7 cells was analyzed with Griess reagent. As seen in Figure 1D, LPS increased the NO concentration up to 51 μM. However, all 24 rosebud extracts at 100 μg/mL displayed NO-inhibitory effects. It was found that Jaemina Red (82.4%), Pretty Velvet (80.4), Hanggina (78.4%), Red Perfume (68.6%), Onnuri (66.7%), Ice Wing (64.7%), and Lover Shy (58.8%) have high NO-inhibitory activity, and in particular, Jaemina Red, Pretty Velvet, and Hanggina were the most effective.

### 3.4. Correlation between Polyphenols and Antioxidative Activities

**Figure 2:**
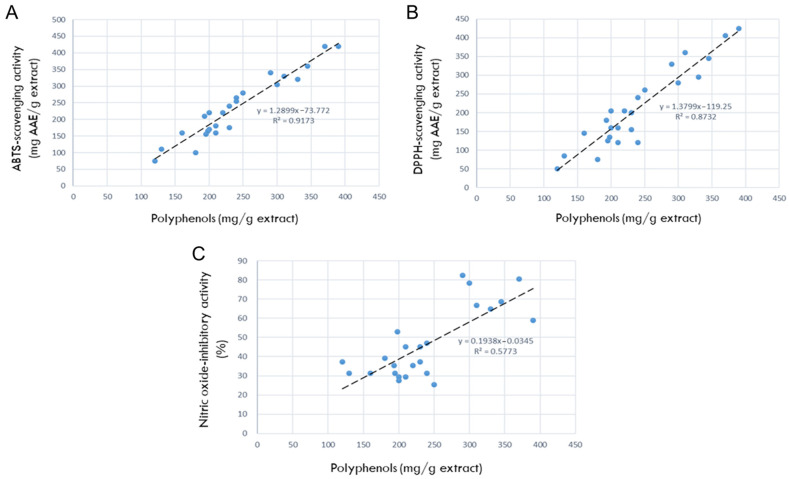
Correlations between antioxidant ingredients in 24 rosebud extracts and their antioxidative or anti-inflammatory activities (100 μg/mL). (**A**) Correlation between polyphenols and 2,2-azino-bis(3-ethylbenzothiazoline 6-sulfonic acid) (ABTS) radical-scavenging activity. (**B**) Correlation between polyphenols and 1,1-diphenyl-2-picrylhydrazyl (DPPH) radical-scavenging activity. (**C**) Correlation between polyphenols and nitric oxide (NO)-inhibitory activities.

The correlations between the antioxidant contents and antioxidant efficacies are shown in Figure 2. As seen in Figure 2A, the correlation between polyphenols and ABTS radical-scavenging activity was R^2^ = 0.9173, indicating a high relationship that the more antioxidants, the better the ABTS-scavenging ability. In parallel with the relationship in ABTS-scavenging potential, the correlation between polyphenols and DPPH radical-scavenging activity was very high (R^2^ = 0.8732), too (Figure 2B).

Therefore, it was confirmed that polyphenols are the main antioxidants in the 24 rosebud extracts, and that among them, Lover Shy, Red Perfume, Onnuri, Jaemina Red, Pretty Velvet, Hanggina, and Ice Wing have the highest antioxidative efficacies.

### 3.5. Correlation between Polyphenols and NO-Inhibitory Activities

The correlation between the antioxidant contents (polyphenols) and NO-inhibition activities was R^2^ = 0.5773 (Figure 2C), indicating a high relationship where the more antioxidants present, the better the NO-inhibitory capacity.

Collectively, Jaemina Red, Pretty Velvet, and Hanggina were found to have the highest anti-inflammatory activity.

### 3.6. Cytotoxicity of PVRE in RAW 264.7 Cells

**Figure 3:**
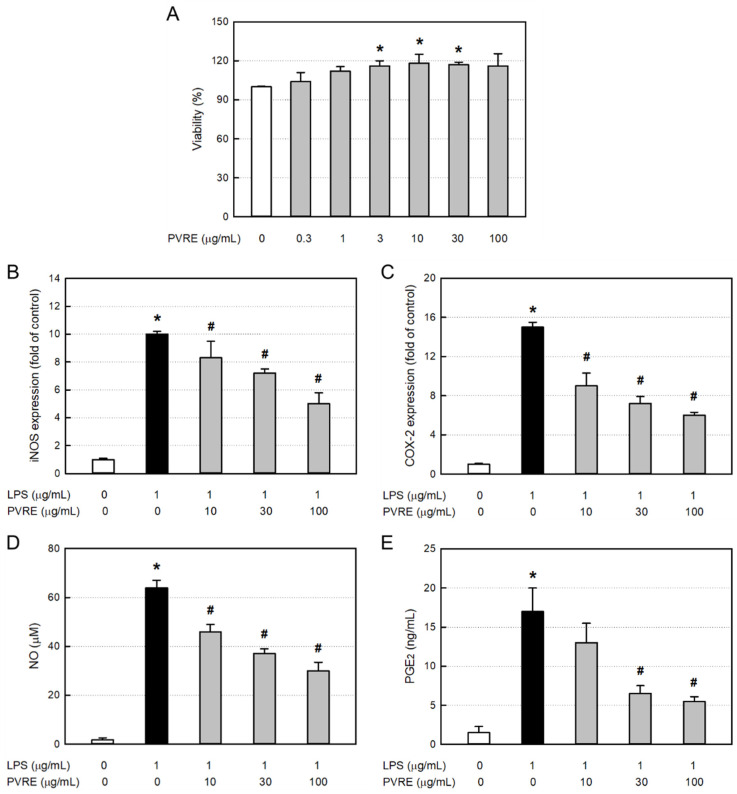
Cytotoxicity and anti-inflammatory activity of Pretty Velvet rosebud extract (PVRE) in RAW 264.7 cells. (**A**) Cytotoxicity of PVRE. (**B**–**E**) Inhibitory activities on inducible nitric oxide synthase (iNOS) expression (**B**), cyclooxygenase-2 (COX-2) expression (**C**), nitric oxide (NO) production (**D**), and prostaglandin E_2_ (PGE_2_) production (**E**) from RAW 264.7 macrophages stimulated with lipopolysaccharide (LPS). * Significantly different from control (*p* < 0.05). ^#^ Significantly different from LPS alone (*p* < 0.05).

In order to assess the cytotoxicity of PVRE itself, RAW 264.7 cells were treated with various concentrations (0.3–100 μg/mL), and 24 h later, the cell viability was measured via MTT assay. PVRE did not exert cytotoxicity up to 100 μg/mL, but rather facilitated proliferation at 10–30 μg/mL (Figure 3A). Accordingly, PVRE was used at non-toxic concentrations of 10, 30, and 100 μg/mL in the following studies for anti-inflammatory activities.

### 3.7. Effects on iNOS and COX-2 Expressions in RAW 264.7 Cells

To elucidate the underlying mechanisms of the anti-inflammatory activity of PVRE, RAW 264.7 cells were treated with LPS (1 μg/mL) and various concentrations (10, 30, or 100 μg/mL) of PVRE, and the mRNA expressions of iNOS and COX-2 were analyzed via qPCR. LPS treatment greatly increased the iNOS mRNA expression (Figure 3B). Such LPS-induced increase in iNOS expression was significantly attenuated by PVRE treatment in a concentration-dependent manner, suggestive of a steroid-like effect. COX-2 mRNA expression in RAW 264.7 cells was also markedly increased after exposure to LPS (Figure 3C). COX-2 expression was down-regulated following treatment with PVRE in a concentration-dependent manner as well, indicating that PVRE possesses an NSAID-like activity.

### 3.8. Effects on NO and PGE2 Production in RAW 264.7 Cells

Inflammatory mediators NO and PGE_2_ produced from LPS-activated RAW 264.7 cells were analyzed with the Griess reagent and ELISA kit, respectively. LPS increased the NO concentration up to 64.0 μM from 1.8 μM in untreated resting cells (Figure 3D). Notably, the LPS-induced production of NO was significantly inhibited by PVRE treatment up to 46.9% of peak value, similar to a steroid’s effect. PGE_2_ production from RAW 264.7 cells was also markedly enhanced after exposure to LPS (Figure 3E). However, such LPS-mediated increase in PGE_2_ production was markedly lowered by treatment with PVRE up to 32.4% of peak value, indicative of an NSAID-like property.

### 3.9. Effect on Exudation in Air-Pouch Inflammation

**Figure 4:**
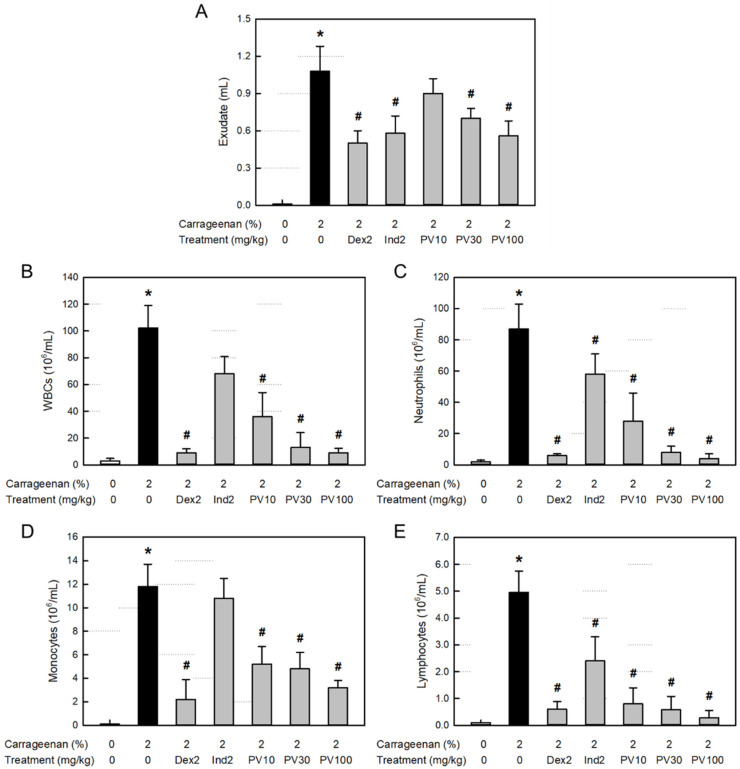
Anti-inflammatory activities of Pretty Velvet (PV) rosebud extract (PVRE) in a dermatitis animal model. (**A**) Effects on λ-carrageenan-induced exudation. (**B**–**E**) Effects on the infiltration of white blood cells (WBCs, (**B**)), neutrophils (**C**), monocytes (**D**), and lymphocytes (**E**) in the exudate. Dex2: 2 mg/kg dexamethasone, Ind2: 2 mg/kg indomethacin, PV10: 10 mg/kg PVRE, PV30: 30 mg/kg PVRE, and PV100: 100 mg/kg PVRE. * Significantly different from control (*p* < 0.05). ^#^ Significantly different from λ-carrageenan alone (*p* < 0.05).

Inflammation causes extravasation of the blood cells and plasma components as well as leakage of tissue fluid. Body fluid transports diverse blood and tissue ingredients, such as proteins, inflammatory cells, and inflammatory mediators, into the inflamed area. As shown in Figure 4A, the λ-carrageenan-induced inflammation greatly increased the exudate volume in the air pouch. However, the increased exudation was potentially attenuated by treatment with dexamethasone (a synthetic steroid) and indomethacin (a representative NSAID). Notably, PVRE inhibited vascular exudation, especially at 100 mg/kg, where it significantly reduced the exudate volume comparable with dexamethasone and indomethacin (2 mg/kg).

### 3.10. Effect on Inflammatory Cells in Exudate

Total WBCs, including neutrophils as well as monocytes and lymphocytes, markedly increased in the exudate from the air-pouch inflammation (Figure 4B–E). The inflammatory cell infiltration was significantly inhibited by both dexamethasone and indomethacin, although dexamethasone was superior to indomethacin. Interestingly, PVRE (10–100 mg/kg) remarkably inhibited the infiltration of all types of inflammatory cells in a dose-dependent manner, notably to the levels achieved with dexamethasone (2 mg/kg) at 30–100 mg/kg.

### 3.11. Effect on Inflammatory Cytokine Concentration in Exudate

**Figure 5:**
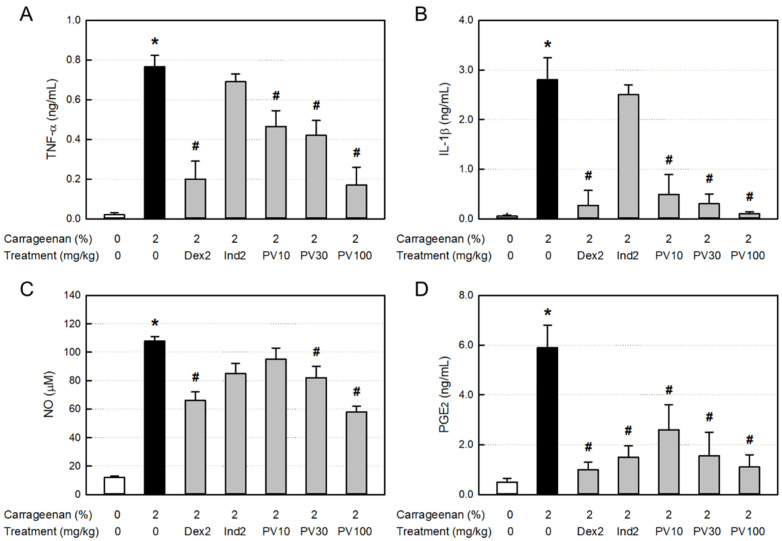
Anti-inflammatory activities of Pretty Velvet (PV) rosebud extract (PVRE) in a dermatitis animal model. (**A**,**B**) Effects on the λ-carrageenan-induced accumulation of tumor necrosis factor-α (TNF-α, (**A**)) and interleukin-1β (IL-1β, (**B**)). (**C**,**D**) Effects on the accumulation of nitric oxide (NO, (**C**)) and prostaglandin E2 (PGE2, (**D**)). Dex2: 2 mg/kg dexamethasone, Ind2: 2 mg/kg indomethacin, PV10: 10 mg/kg PVRE, PV30: 30 mg/kg PVRE, and PV100: 100 mg/kg PVRE. * Significantly different from control (*p* < 0.05). ^#^ Significantly different from λ-carrageenan alone (*p* < 0.05).

The major inflammatory cytokines TNF-α and IL-1β in the inflamed exudate greatly increased (Figure 5A,B). Both the cytokines increased by the λ-carrageenan challenge were significantly inhibited only by dexamethasone, but not by indomethacin. Notably, TNF-α and IL-1β were also inhibited by PVRE in a dose-dependent manner, suggestive of the steroid-like activity of PVRE.

### 3.12. Effects on NO and PGE_2_ Concentrations in Exudate

In parallel with the inflammatory cytokines, the concentrations of NO and PGE_2_ in the exudate following λ-carrageenan exposure markedly increased (Figure 5C,D). The NO accumulation was attenuated by treatment with dexamethasone. As inferred from the inhibition of cytokines, the NO was also significantly suppressed by PVRE. Although TNF-α, IL-1β, and NO were not affected by indomethacin, PGE_2_ was remarkably inhibited by both dexamethasone and indomethacin. It was also lowered by PVRE in a dose-dependent manner, suggesting that PVRE has both steroid- and NSAID-like activities.

### 3.13. Effect on Blood Vessel Area

**Figure 6:**
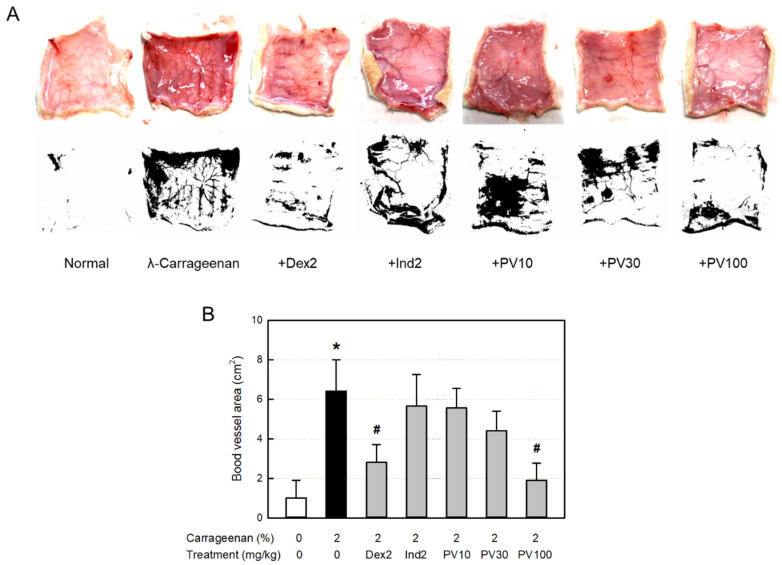
Anti-inflammatory activities of Pretty Velvet (PV) rosebud extract (PVRE) in a dermatitis animal model. (**A**) Representative findings of λ-carrageenan-induced erythema (vascular swelling) in the skin (upper: gross findings, lower: ImageJ findings). (**B**) Blood vessel area analyzed with ImageJ. Dex2: 2 mg/kg dexamethasone, Ind2: 2 mg/kg indomethacin, PV10: 10 mg/kg PVRE, PV30: 30 mg/kg PVRE, and PV100: 100 mg/kg PVRE. * Significantly different from control (*p* < 0.05). ^#^ Significantly different from λ-carrageenan alone (*p* < 0.05).

Inflammation occurs along with vasodilatation, leading to erythema and edema. The dermal blood vessel dilatation was observed in the skin (Figure 6A). Accordingly, the blood vessel area measured with ImageJ increased more than six times, which was significantly attenuated by dexamethasone (Figure 6B). Interestingly, PVRE (100 mg/kg) exhibited a potential effect similar to that of dexamethasone (2 mg/kg).

### 3.14. Microscopic Findings

**Figure 7:**
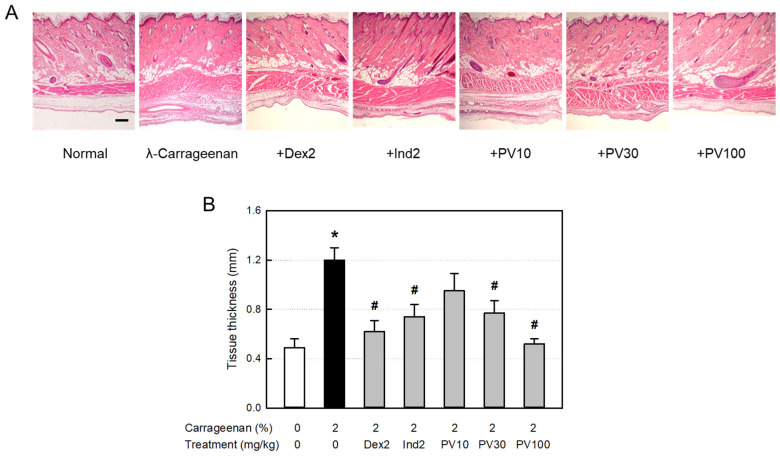
Anti-inflammatory activities of Pretty Velvet (PV) rosebud extract (PVRE) in a dermatitis animal model. (**A**) Representative microscopic findings of λ-carrageenan-induced edema (tissue thickening) and inflammatory cell infiltration in the skin. (**B**) The thickness of air-pouch lining tissue. Dex2: 2 mg/kg dexamethasone, Ind2: 2 mg/kg indomethacin, PV10: 10 mg/kg PVRE, PV30: 30 mg/kg PVRE, and PV100: 100 mg/kg PVRE. * Significantly different from control (*p* < 0.05). ^#^ Significantly different from λ-carrageenan alone (*p* < 0.05)**.** Scale bar = 200 μm.

From the histopathological examination of the tissues surrounding the λ-carrageenan-exposed air pouches, severe infiltration of many inflammatory cells into the dermal and subcutaneous tissues was observed (Figure 7A). In addition, blood vessel dilatation, as well as edema in the dermal and muscular layers, were also seen, as confirmed by the thickness of the lining and muscle layers (Figure 7B). The inflammatory responses were inhibited by dexamethasone and indomethacin. Notably, PVRE also exerted strong anti-inflammatory activity in a dose-dependent manner, wherein PVRE’s effect at 100 mg/kg was comparable to dexamethasone and superior to indomethacin (2 mg/kg).

## 4. Discussion

Most of the animals consuming oxygen for metabolism suffer from tissue damage, called oxidative stress, and aging due to the generation of ROS [43]. Organisms synthesize or ingest antioxidants in the body to block cell and tissue damage caused by ROS. If oxidative stress in the body goes beyond the antioxidant defense capacity that counteracts it, supplementation with antioxidants can theoretically limit oxidative damage. As such, the search for a compound capable of scavenging ROS or a substance that inhibits the formation of oxides is being actively conducted. Among the possible phytochemicals known, polyphenols, for instance, have an important antioxidant bioactivity, but other compounds can also be enrolled for this possible effect as carotenoids, some kind of alkaloids, organosulfur derivatives, etc. [30].

ABTS and DPPH-scavenging activities are common tools for the rapid assessment of the antioxidative potentials of diverse natural products [44]. In the present study, the polyphenol contents and antioxidative activities of 24 rosebud extracts showed high relationships, and among them, Lover Shy and Pretty Velvet were found to be the highest in both antioxidant ingredients and their activities.

Tissue damage caused by ROS is involved in the most inflammatory responses. The inflammation process consists of a cascade of cellular as well as microvascular reactions. Therefore, the anti-inflammatory effects of the 24 rosebud extracts were assessed in LPS-activated RAW 264.7 macrophages, from which Pretty Velvet, Jaemina Red, and Hanggina displayed the highest NO-inhibitory activities. Thus, PVRE, possessing the highest antioxidative and anti-inflammatory potentials, was selected as a candidate for further studies on therapeutic effects and underlying mechanisms in vitro and in vivo. By comparison with high correlations between polyphenol content and antioxidative activity, the correlation between polyphenols and anti-inflammatory activity was relatively low (R^2^ = 0.5773). Such results indicate that additional ingredients, such as terpenes, might be involved in the anti-inflammatory effects. Indeed, it was demonstrated that certain terpenes could reduce inflammatory responses by decreasing the release of pro-inflammatory cytokines mediated by nuclear transcription factor-κB [45].

The process of inflammation is accompanied by exudation, infiltration of inflammatory cells, and the production of cytokines and mediators. The accumulation of circulating leukocytes and monocytes, followed by lymphocytes, is one of the most visible phenomena of inflammation [6,46]. In this process, chemo-attraction by resident cells plays an important role in inducing inflammatory cell recruitment to the damaged sites [47]. Inflammatory cytokines are produced predominantly by activated macrophages, and mediate upregulation of inflammatory reactions [48]. In particular, certain inflammatory cytokines such as TNF-α and IL-1β are involved in the process of pathologic pain [49,50]. Although NO produced by constitutive NOS (cNOS) plays an important role in diverse physiological functions, excessive production of NO from cytokine- or bacterial endotoxin-activated iNOS causes acute pain, edema, and even shock [51,52]. It has been well defined that the TNF-α–iNOS–NO pathway is controlled by steroids [9,10]. Interestingly, PVRE strongly inhibited the iNOS mRNA expression and the ensuing NO production in LPS-stimulated RAW 264.7 macrophages. From such phenomena, it is inferred that PVRE has a steroid-like action mechanism.

PGs produced from AA by enzyme COX-1 are important physiological intrinsic regulators for blood blow, gastric mucosal protection, and renal and uterine functions. Among diverse PGs, PGE_2_ synthesis is affected by the expression of COX-2, levels of which are highly inducible in many tissues by inflammatory factors, including cytokines and growth factors. Indeed, PGE_2_ is an important mediator of inflammatory and immune responses during acute and chronic infections [53]. By comparison with the TNF-α–iNOS–NO pathway, the AA–COX-2–PGE_2_ pathway is regulated by NSAIDs. In the present study, the COX-2 expression and PGE_2_ production were markedly inhibited by PVRE, indicative of an NSAID-like activity of PVRE. Based on the dual effects of PVRE on NO and PGE_2_ regulation, it was expected that PVRE could be a potential candidate for anti-inflammation. So, further studies in vivo were conducted.

In in vivo air-pouch inflammation, λ-carrageenan caused vascular dilatation (erythema) and dermal edema, resulting in a great increase in exudate volume, which are indicative of vascular leakage of serum components [54]. The vasodilatation and exudation were effectively attenuated by dexamethasone (a steroid), indomethacin (an NSAID), and PVRE. In addition, the huge increases in inflammatory cells (WBCs, neutrophils, monocytes, and lymphocytes), cytokines (TNF-α and IL-1β), and NO in the exudate was also markedly inhibited by dexamethasone and PVRE, but not by indomethacin. It is of interest to note that λ-carrageenan induces inflammatory cell infiltration and activation to release cytokines, iNOS expression, and NO production, and that PVRE blocks the cell chemo-attraction, activation, and iNOS expression similarly to the steroids’ activity.

λ-Carrageenan greatly increased the PGE_2_ concentration in the exudate, indicating the activation of the COX-2 inflammatory pathway, which was inhibited by indomethacin as well as PVRE. Therefore, it was confirmed that PVRE has both steroid- and NSAID-like activities. Separately, the PGE_2_-reducing activity of dexamethasone may be due to the inhibitory effect of steroids on the arachidonic acid-degrading phospholipase A_2_ (PLA_2_) [55].

The anti-inflammatory effects of PVRE on vascular exudation, edema, and inflammatory cell infiltration were confirmed in microscopic examinations. The dermal tissues exposed to λ-carrageenan exhibited severe vasodilatation, increasing the blood vessel area under ImageJ analysis, as well as tissue edema, increasing the dermal and muscular layer thickening. In addition, severe infiltration of inflammatory cells was observed in the dermal and subcutaneous tissues. Interestingly, such inflammation was substantially suppressed by PVRE and dexamethasone, which were superior to indomethacin. Such phenomena indicate that λ-carrageenan-induced vascular response and inflammatory cell infiltration are mainly mediated by the TNF-α–iNOS–NO pathway.

From the present study results, several rosebud extracts were found to contain high concentrations of antioxidant ingredients, and exhibited close relationships with antioxidative and NO-inhibitory activities. At the same time, PVRE inhibited inflammatory reactions in RAW 264.7 macrophages by down-regulating iNOS and COX-2 mRNA expression. In addition, PVRE decreased the λ-carrageenan-induced skin inflammation in vivo: i.e., it blocked vascular leakage, inflammatory cell infiltration, and the production of inflammatory cytokines and mediators in dual mechanisms on the TNF-α–iNOS–NO and arachidonic acid–COX-2–PGE_2_ pathways, which are controlled by steroids and NSAIDs, respectively.

Therefore, the results indicate that rosebud extracts containing large amounts of antioxidants, including PVRE, from newly crossbred roses could be candidates for the development of antioxidative and anti-inflammatory functional products. In addition, it is expected that novel medicinal plants through crossbreeding could be produced in the near future.

## Figures and Tables

**Table 1:** Primer sequences used in this study.

Genes	Sequence 5′-3′	Temperature
iNOS	Forward: CAGGATCCAGTGGTCCAACC Reverse: CGTACCGGATGAGCTGTGAA	60 °C 59 °C
COX-2	Forward: GTACAAGCAGTGGCAAAGGC Reverse: ACGAGGTTTTTCCACCAGCA	60 °C 60 °C
GAPDH	Forward: GACCTCATGGCCTACATGGC Reverse: GCCCCTCCTGTTATTATGGGG	60 °C 59 °C

iNOS: inducible nitric oxide synthase, COX-2: cyclooxygenase-2, and GAPDH: glyceraldehyde 3-phosphate dehydrogenase.

## Data Availability

Data are contained within the article.
